# Odontogenic MSC Heterogeneity: Challenges and Opportunities for Regenerative Medicine

**DOI:** 10.3389/fphys.2022.827470

**Published:** 2022-04-19

**Authors:** Yuan Chen, Zhaoyichun Zhang, Xiaoxue Yang, Anqi Liu, Shiyu Liu, Jianying Feng, Kun Xuan

**Affiliations:** ^1^ State Key Laboratory of Military Stomatology, National Clinical Research Center for Oral Diseases, Department of Preventive Dentistry, School of Stomatology, The Fourth Military Medical University, Xi’an, China; ^2^ School of Stomatology, Zhejiang Chinese Medical University, Hangzhou, China

**Keywords:** heterogeneity, mesenchymal stem cells, odontogenic, development, regeneration

## Abstract

Cellular heterogeneity refers to the genetic and phenotypic differences among cells, which reflect their various fate choices, including viability, proliferation, self-renewal probability, and differentiation into different lineages. In recent years, research on the heterogeneity of mesenchymal stem cells has made some progress. Odontogenic mesenchymal stem cells share the characteristics of mesenchymal stem cells, namely, good accessibility, low immunogenicity and high stemness. In addition, they also exhibit the characteristics of vasculogenesis and neurogenesis, making them attractive for tissue engineering and regenerative medicine. However, the usage of mesenchymal stem cell subgroups differs in different diseases. Furthermore, because of the heterogeneity of odontogenic mesenchymal stem cells, their application in tissue regeneration and disease management is restricted. Findings related to the heterogeneity of odontogenic mesenchymal stem cells urgently need to be summarized, thus, we reviewed studies on odontogenic mesenchymal stem cells and their specific subpopulations, in order to provide indications for further research on the stem cell regenerative therapy.

## Introduction

Cellular heterogeneity was first discovered in tumor cells, and the term is used to describe the differences in genes and phenotypes between different tumor cells (Ono et al., 2021; Pe’er et al., 2021). In terms of pluripotency, self-renewal ability, and other traits, mesenchymal stem cells (MSCs), as undifferentiated cells, show similar behaviors to tumor cells, as mentioned in many studies; in contrast, the heterogeneity of MSCs has rarely been reported (Hayashi et al., 2019). Initially, it was found that alkaline phosphatase (ALP) is highly expressed in somes but not other individual colony cultures of MSCs, and there is also a third type that has intermediate expression; in addition, this factor is not expressed in the periphery (Friedenstein et al., 1982). Further research showed that the expression patterns of markers such as CD90 and CD45 in several types and subpopulations of MSCs are quite different (Abe et al., 2008; Li et al., 2012; Mabuchi et al., 2021). With the help of new technologies, such as single-cell RNA sequencing (scRNA-seq) and its derivative technologies, a better approach for studying cell heterogeneity has been developed (Chen S et al., 2020; Lei et al., 2021). Using single-cell sequencing, it was found that even within an MSC subpopulation, due to changes in the internal and the external factors, the gene expression also differs among cells (Bianco et al., 2001; Mabuchi et al., 2021). Overall, heterogeneity of the MSC phenotype is likely to exist among cell lines, among cells within a line, and among temporal states of a single cell.

Odontogenic MSCs have been isolated from dental tissue, including dental pulp (Gronthos et al., 2000), exfoliated deciduous teeth (Miura et al., 2003), periodontal ligaments (Seo et al., 2004), dental follicles (Morsczeck et al., 2005), apical papillae (Sonoyama et al., 2006), papillae (Tziafas and Kodonas, 2010) and periapical cysts (Marrelli et al., 2013). Odontogenic MSCs have become an attractive source of autologous MSCs because they exhibit the characteristics of vasculogenesis and neurogenesis (Liu et al., 2015), along with their good accessibility, low immunogenicity and high stemness (Merimi et al., 2021). There has been some clinical evidence verifying the therapeutic effect of odontogenic MSCs in tissue regeneration. For example, implantation of human deciduous pulp stem cells (hDPSCs) into injuried incisors can promote pulp regeneration and partial tooth recovery (Xuan et al., 2018). However, the effect of odontogenic MSCs on tissue regeneration and disease management remains unclear (Levy et al., 2020). It is generally accepted that the extracellular environment affects the therapeutic efficacy of engrafted odontogenic MSCs. A chronic inflammatory environment interferes with odontogenic MSC osteogenesis, resulting in osteolysis (Bressan et al., 2019).

Recently, the heterogeneity of odontogenic MSCs has attracted attention. To date, the sudy and application of human MSCs are mostly based on the hybrid effect, and only a small part of the hybrid may play a pivotal role in regeneration and immunoregulation (Huang et al., 2019). In this review, the characteristics and application prospects of odontogenic MSCs are summarized according to their specific subpopulations, with the aim of obtaining a better understanding the opportunities and challenges generated by the heterogeneity of stem cells in the context of regenerative medicine and providing ideas for further research on the stem cell regenerative therapy.

## MSC Heterogeneity During Development and Regeneration

MSCs, which can alternatively be defined as multipotent MSCs, are a series of premature stromal cells with multilineage differentiation potential. They can be amplified *in vitro* and differentiate into specific tissues (such as bone, cartilage, adipose tissue, etc.) under diverse conditions (Uccelli et al., 2008). From almost every type of connective tissue, specific MSCs can be isolated, which explains the importance of MSCs in tissue regeneration research. However, researchers have suggested that there are differences within a single type of MSCs derived from different individuals (Lv et al., 2014); either different cultivation conditions or different numbers of cell passages can determine gene or protein expression profiles, which directly reflects the phenotype of the colony. This is currently called MSC heterogeneity.

For example, in skin tissue, there are many specialized mesenchymal cells called dermal papillae (DPs) located at the base of the hair follicles, which are essential for hair regeneration (Driskell et al., 2011; Morgan, 2014). Freshly isolated DPs or in vitro-cultured DP cells can induce *de novo* hair follicle formation when implanted elsewhere, with the hair type determined by the source of the DPs (Jahoda et al., 1984). This indicates that DP cells are intrinsically heterogeneous and contain hard-wired positional memory that in turn instructs the overlaying epithelial cell activity. Furthermore, the same holds for bone marrow MSCs; only a fraction of colony-forming units from plastic adherence isolated MSCs(PA-MSCs) exhibited multipotency, indicating that PA-MSCs comprise a heterogeneous population of cells with different lineage commitments, which may be relate to their *in vivo* environments (Lv et al., 2014). This is reflected in the differences in the protein expression profiles, cytokine profiles, and differentiation potency of MSCs from various source. In addition, the oral cavity is a source of MSCs, but the heterogeneity of these MSCs has not been systematically reviewed.

## Odontogenic MSC Heterogeneity

The mesoderm and neural crest are currently considered to be the main sources of MSCs (Takashima et al., 2007). Mesoderm-derived MSCs mainly give rise to bone and connective tissue. When cultured *in vitro*, they show potential for chondrogenic, osteogenic and adipogenic differentiation (Vodyanik et al., 2010). Neural crest-derived MSCs are considered to generate the craniofacial bones. Notably, they show neurogenic potential compared to mesoderm-derived MSCs (Isern et al., 2014).

Odontogenic MSCs are considered to be derived from the neural crest (Thesleff and Nieminen, 1996) and have become a remarkable source of autologous MSCs. They have been isolated and identified from various sites in dental tissues, including apical papillae, exfoliated deciduous teeth, dental follicles, periodontal ligaments, dental pulp, dental papillae and periapical cysts. In 2000, Gronthos et al. isolated a clonogenic group of dental pulp cells that have similar capabilities as bone marrow stromal cells (BMSCs), including self-renewal ability and multidirectional differentiation potential (Gronthos et al., 2000). Since then, an increasing number of MSCs have been isolated from dental pulp, apical papillae, dental follicles, periodontal ligaments, periapical cysts and even the gingiva (Zhang et al., 2009). These odontogenic MSCs have been widely used in various tissue regeneration studies, including studies of bone, nerve and liver regeneration. Several studies have found that in animal calvarial bone defect models, odontogenic MSCs promote new bone formation and play an important role in bone regeneration (Yang H et al., 2019). In the context of neural restoration, odontogenic MSCs can differentiate into neuron-like cells and achieve neuro-regeneration, and their effectiveness in the treatment of ischemic vascular diseases has been validated (Zhang et al., 2018; Gonmanee et al., 2020). Moreover, odontogenic MSCs have the potential to differentiate into hepatocyte-like cells. Transplantation of stem cells from exfoliated deciduous teeth (SHED) has been reported to significantly reduce rat liver fibrosis and normalize the disordered liver structure (Yokoyama et al., 2019). Notably, compared with MSCs from other sources, odontogenic MSCs exhibit several unique advantages.

**FIGURE 1:**
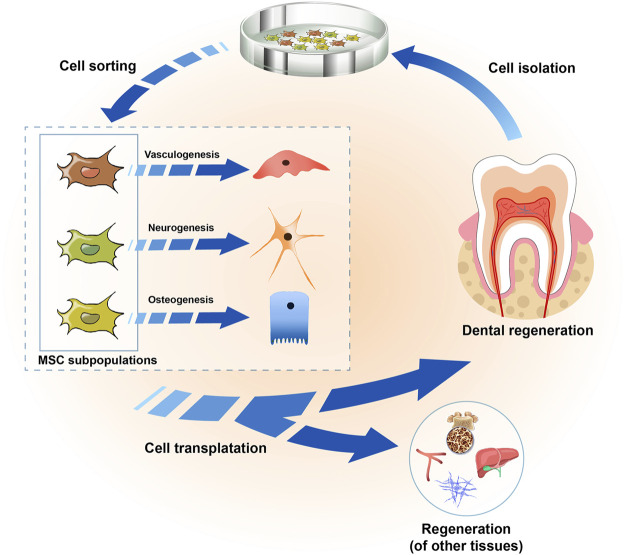
Strategies for utilizing the odontogenic MSC heterogeneity in tissue regeneration. Odontogenic MSCs were isolated from dental tissue, including dental pulp, periodontal ligaments, dental follicles, apical papillae, and the gingiva, and they showed heterogeneity in terms of their pluripotency *in vitro* culture. They could be sorted into several cell subpopulations according to their characteristics, with enhanced vasculogenic, neurogenic and osteogenic differentiation abilities. In the regeneration of teeth or other tissues, such as bone, liver, nerves and blood vessels, the selection of one or more cell subpopulations according to different needs might improve the outcomes of regenerative medicine.

Interestingly, there seem to be location-related differences in the regenerative potential of these cells. For example, stem cells from periodontal ligaments can differentiate into cementoblasts (J. W. Yang et al., 2020) and concentrate collagen fibers similar to Sharpey fibers (Hasegawa et al., 2005), whereas stem cells from dental pulp show the ability to produce calcified nodules, the components of which are similar to dentin (Zhang et al., 2005; Feng et al., 2011). In this regard, the functional heterogeneity of MSCs makes the application of MSCs more challenging, as different cells may show different therapeutic effects for different therapeutic purposes. However, this heterogeneity also raises the opportunity that dividing MSCs into different subgroups according to their different functions may improve the outcomes of stem cell therapy (Figure 1). A study proved that after the transplantation of dental pulp stem cells (DPSCs) *in situ*, the vascularized pulp tissue of canine teeth was successfully repaired (Iohara et al., 2013). Similarly, stem cells of the apical papillae (SCAPs) have been shown to exhibit the ability to support tooth root formation (Abe et al., 2008). On the other hand, gingival mesenchymal stem cells (GMSCs) injected through the tail vein could accurately locate periodontal injury sites and participate in periodontal tissue regeneration (Sun et al., 2019), whereas DPSCs could hardly repair periodontal tissue defects (Park et al., 2011). Nevertheless, some studies suggested that DPSCs might be able to accomplish periodontal tissue repair and regeneration (Liu et al., 2011). These results suggest that investigations of the role of different odontogenic MSC subpopulations in tissue repair and regeneration should be carried out more systematically.

The cellular environment required for growth and development is highly dynamic and heterogeneous. The cellular origin of the tooth can be shown by using lineage tracking, which helps to better explore the cellular environment required for tooth development. In mouse incisors, the population of peripheral glial MSCs originates trom the teeth, and these cells can differentiate into odontoblasts and dental pulp cells (Kaukua et al., 2014; Krivanek et al., 2020). In addition, non-glial-derived dental MSCs have been reported in mouse incisors, and these cells are found in the neurovascular bundle and responsive to Shh (Zhao et al., 2014). Lineage tracing of these cells using Ng2-Cre showed that they are derived from pericytes and mainly act on the vascular system. They are also responsible for the continuous generation of odontoblasts, especially after odontoblasts are damaged (Feng et al., 2011; Zhao et al., 2014; Pang et al., 2016). Taken together, these findings indicate that more than one type of MSC is involved in tooth development, and different cells perform different functions.

## Advantages of Odontogenic MSCs in Tissue Regeneration

### Better Accessibility

Although MSCs can be isolated from connective tissue throughout the body, the invasiveness of the collection process often limits their clinical application. In contrast, odontogenic MSCs, which can be isolated from discarded teeth and periodontal tissue, are easier to obtain from the body (J. Yang et al., 2020). Teeth extracted due to impaction, trauma or severe periodontitis and exfoliated deciduous teeth that have fallen out naturally, which are often abandoned, can be a rich source of odontogenic MSCs (Chen et al., 2012). In addition, gingiva that was removed for aesthetic or pathological reasons can also be an attractive source of MSCs. In comparison to the difficulty of obtaining stem cells from other organs or tissues in the human body, not only is it easy to access to the collection site of odontogenic MSCs but also the extraction of stem cells from dental tissue is highly efficient.

### Lower Immunogenicity

Low immunogenicity has been reported to be a common characteristic among different MSC populations, including odontogenic MSCs (Laing et al., 2018). This is mainly because they do not express immune costimulatory factors, such as major histocompatibility complex class II antigen, as well as CD40, CD80 and CD86 (Wada et al., 2013). In this regard, the low immunogenicity of odontogenic MSCs solves the problem of transplant rejection caused by immune incompatibility between the donor and the recipient. Transplant rejection usually occurs after transplantation of allogeneic cells, tissues or organs, which is one of the critical issues in tissue engineering. Of note, odontogenic MSCs have immunomodulatory properties that inhibit immune responses and make them suitable for the treatment of autoimmune and inflammation-related diseases (Le Blanc, 2006). For example, PDLSCs can induce macrophages to polarize toward the M2 phenotype that supports tissue repair, thereby changing the immune microenvironment and promoting periodontal regeneration (Liu et al., 2019). *In vitro* experiments showd that DPSCs could reduce the viability of natural killer cells and inhibit their cytotoxic activity. Moreover, SHEDs increase the ratio of regulatory T cells (Tregs) to T helper 17 (Th17) cells *in vivo* by restraining the differentiation of Th17 cells (Yamaza et al., 2010). In conclusion, the immunomodulatory properties of odontogenic MSCs allow them to support allotransplantation for tissue-engineering applications, addressing the problem of failure to generate sufficient cells from a single donor for cell transplantation therapy.

### Higher Stemness

Odontogenic MSCs show higher stemness because tooth development continues until the permanent teeth replace the deciduous teeth after birth. DPSCs and SHEDs have been reported to have superior proliferative capability to BMSCs and to express some pluripotent markers, such as Sox-2, Nanog and Oct-3/4 (Monterubbianesi et al., 2019; Yang X et al., 2019). GMSCs have also been shown to maintain long-term proliferation with a faster population doubling time than BMSCs (Al Bahrawy et al., 2020). In terms of osteogenic differentiation, DPSCs showed higher odontogenic capacity than BMSCs (Monterubbianesi et al., 2019). After tooth damage occurs, DPSCs are quickly activated, and then proliferate, migrate and differentiate into odontoblasts, replacing apoptotic odontoblasts (Chen et al., 2016). Moreover, from a developmental point of view, teeth and periodontal tissue are produced by the continuous interaction between ectodermal epithelial cells and ectodermal mesenchymal cells from the neural crest and mesoderm (Kota et al., 2016). All subpopulations of odontogenic MSCs not only have the general characteristics of other MSCs but also have neurogenic abilities similar to those of neural crest-derived stem cells (Liu et al., 2015). Several odontogenic MSC subpopulations have been shown to be able to differentiate into nerve cells, including SHEDs (Rosa et al., 2016), dental follicle stem cells (DFSCs) and SCAPs (De Berdt et al., 2015) and human periapical cyst-mesenchymal stem cells (hPCy-MSCs) (Tatullo et al., 2020). Notably, SCAPs can express nestin, GAD, βIII tubulin and neurofilament M and other neuronal cell-associated markers, as well as some neurotrophic factors or neuroprotective factors, to promote neurogenesis and regeneration (de Almeida et al., 2014). Overall, odontogenic MSCs show higher stemness than BMSCs, suggesting that they might play key roles in periodontal regeneration and tooth homeostasis.

Although odontogenic MSCs exhibit many advantages, as described above, they are heterogeneous. The phenotype and genotype of each cell determine its functions. Therefore, studying cell heterogeneity will enable accurate targeting and improved effects on tissue regeneration.

## Characteristics of Several Odontogenic MSC Subpopulations

### Odontogenic MSC Subpopulations

**FIGURE 2:**
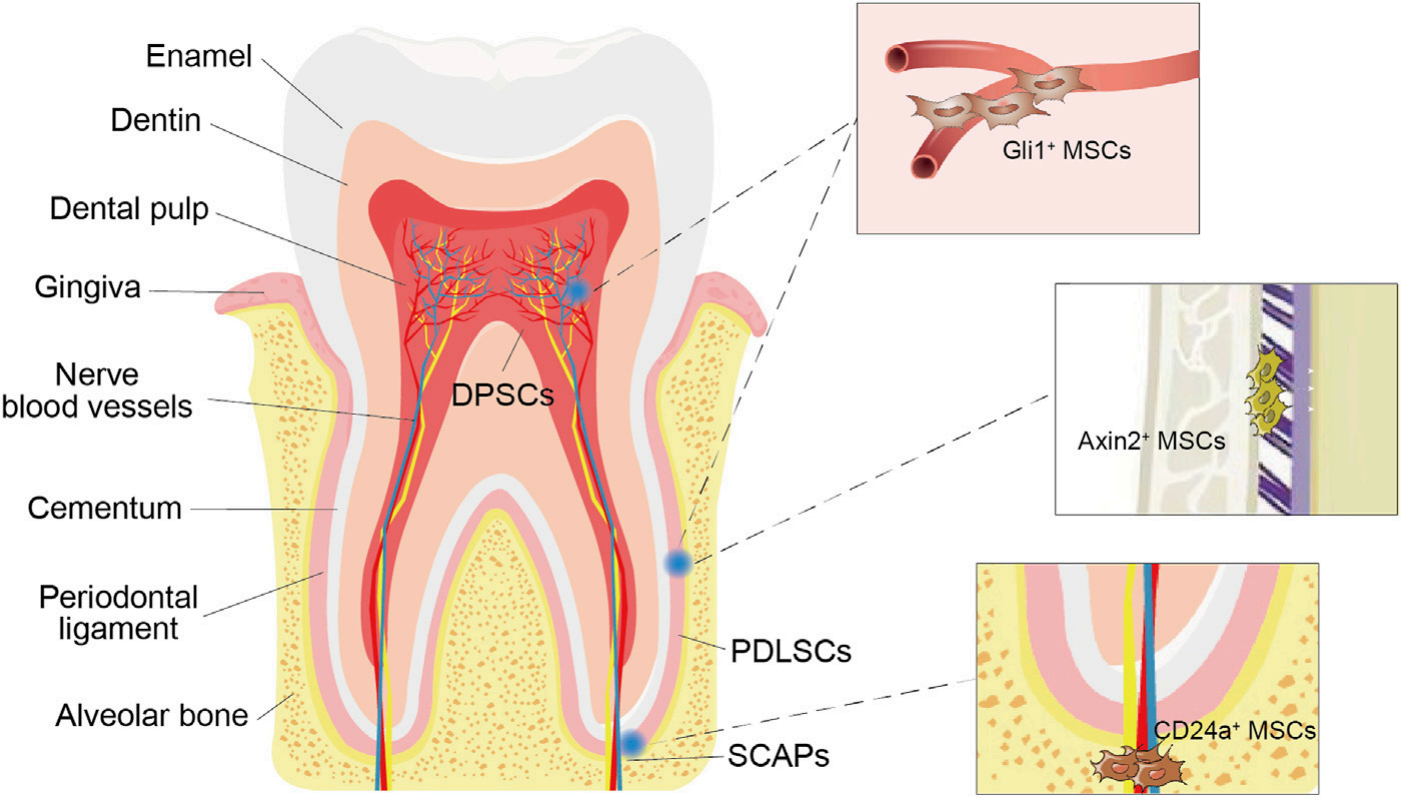
Odontogenic MSCs and their subpopulations. Various types of stem cells, such as dental pulp stem cells (DPSCs), periodontal ligament stem cells (PDLSCs) and stem cells from the root apical papilla (SCAPs), can be found in the dental pulp and periodontal ligament around the tooth root. Gli1^+^ MSCs surround the vasculature exclusively. Axin2^+^ MSCs were identified in the periodontal ligament (PDL). CD24a + MSCs were present in human dental papilla tissues.

With the improving understanding of the heterogeneity of MSCs, an increasing number of odontogenic MSC subpopulations have been isolated (Figure 2). CD24^+^ cells were isolated from apical papilla and identified as undifferentiated SCAPs with greater dentine regeneration capacity (Sonoyama et al., 2006). A group of Gli1^+^ MSCs were also isolated from the periodontal ligaments of mouse molars, surrounding the neurovascular bundle and forming the PDL, cementum, and alveolar bone (Men et al., 2020). In addition, cell lineage tracing studies have shown that the rapid proliferation of Axin2^+^ MSCs and their progeny can directly contribute to the cellular cementum and the postnatal acellular cementum (Xie et al., 2019). Overall, these subgroups have distinct characteristics and biological activity and enhance the regenerative potential of certain tooth structures. Therefore, it is necessary to further subdivide odontogenic stem cells, clarify their differences in differentiation potential, proliferation rate, immunosuppression ability and other biological functions, and conduct comparative studies to standardize treatment methods (Table 1).

**TABLE 1:** Summary of main characteristics and therapeutic efficacy of MSC subpopulations.

**MSC Subpopulations**	**Tissue Sources**	**Primary Function**	**Main characteristics**	**Therapeutic Efficacy**
**Gli1** ^**+**^ **MSC**	Mouse incisor	Tooth support	Strong mobilization in response to tissue injury	Periodontal bone regeneration ↑
		Dentin formation	Generating bone matrix *in vivo*	Fracture repair ↑
**Axin2** ^**+**^ **MSC**	Periodontal ligament	Tooth support	Give rise to cementoblasts and cementocytes *in vivo*	Cementum regeneration ↑
		Cementum formation	Express transcription factors for controlling cementum formation	
**CD24a** ^**+**^ **MSC**	Apical papilla	Tooth support	Higher proliferation capacity than BM-MSCs	Dental pulp regeneration ↑
		Wound healing	Enhanced osteogenic/odontogenic differentiation capabilities	
**CD146** ^**+**^ **MSC**	Periodontium and pulpal tissue of deciduous teeth	Dentin formation	Express neurogenic markers	Periodontal bone regeneration ↑
			Form dentin-like structures *in vivo*	Dental pulp regeneration ↑
			Higher proliferation capacity than DPSCs and BM-MSCs	
**Stro-1** ^**+**^ **MSC**	Dental pulp of permanent teeth	Dentin formation	Generate the cementum-PDL structure *in vivo*	Periodontal tissue regeneration ↑
			Higher proliferation capacity than DPSCs and BM-MSCs	Alveolar bone regeneration ↑
**CD105** ^**+**^ **MSC**	Dental pulp of permanent teeth	Dentin formation	Express neurogenic markers	Dental tissue inflammation ↓
			Higher proliferation capacity and odontogenic capacity than BM-MSCs	Dentin regeneration ↑
				Dental pulp regeneration ↑
				Alveolar bone regeneration ↑
				Osteogenic potential ↑

### Gli1^+^ MSCs and Their Role in Odontogenic Regeneration

Gli1 is one of three GLI family transcription effectors at the terminal end of the hedgehog signaling (Hh) pathway, which participate in the progression of cell differentiation and development. Gli1 was first identified in human glioblastoma (Kinzler et al., 1987). Later, in the heart, lung, kidney and other tissues, it was found to have different functions and to participate in injury repair (Kramann et al., 2015). Gli1^+^ cells surround the vasculature exclusively and are negative for the expression markers of neural or glial cells (b3-tubulin, S100), endothelium (GS-IB4), smooth muscle cells (aSMA), or pericytes (NG2, LepR) (Zhao et al., 2014; Men et al., 2020). Therefore, Gli1^+^ cell sorting can improve the purity of MSCs and reduce the impact of impurities on clinical applications.

Gli1^+^ cells have been identified as the stem cells of the mouse long bone, craniofacial bones, and incisor (Pang et al., 2015; Zhao et al., 2015; Shi et al., 2017). It was found that inhibition of Gli1^+^ cells in the mouse incisor can result in alveolar bone resorption and wider PDL gaps in the periodontium of mice (Chen S et al., 2020), suggesting that the activation of Gli1^+^ cells may be related to periodontal development and repair in mice.

A large body of work has indicated that Hh critically regulates osteoblast differentiation and is implicated in alveolar bone development and remodeling. As a transcription effector at the terminal end of the Hh signaling pathway, Gli1 may be a marker of a specific MSC subpopulation with enhanced osteogenic capacity (Shi et al., 2017). Several studies have investigated the distribution characteristics and physiological functions of Gli1^+^ cells *in vivo*. Gli1 expression was detected by using Gli1-LacZ mice, and it was found that there was a large number of stem cells in the periodontal membrane of young mice, especially in 1/3 of the root tip. Gli1^+^ cells differentiate and mature during the development of the periodontal membrane, and some fusiform or polygonal fibroblasts develop into long fusiform cells, which account for a large proportion of the total number of periodontal ligament cells (Men et al., 2020). Of note, Gli1^+^ cells in the periodontal membrane can gradually differentiate and proliferate into osteocytes around alveolar bone, and their numbers increase gradually, with the secretion of bone matrix and the formation bone (Hosoya et al., 2020; Liu et al., 2020). Taken together, these findings indicate that Gli1^+^ cells may be the main cell source for the periodontal membrane and alveolar bone and participate in growth and development during the peak period.

Gli1^+^ cells exhibit strong mobilization in response to tissue injury. These cells proliferated massively and migrated to the damaged area 24 h after tooth injury and formed repaired dentin later (Zhao et al., 2014). Consistently, 10 days after fracture, Gli1^+^ cells were detected in the whole fracture callus, indicating that Gli1^+^ cells, as progenitors of osteoblasts, are responsible for normal bone formation as well as fracture repair (Shi et al., 2017). The ability of Gli1^+^ cells to respond quickly to injury of multiple tissues and organs suggests that they are a specific MSC subpopulation with potential for morphologic development and repair and regeneration.

Although Gli1^+^ cells are negative for markers of neural or glial cells (b3-tubulin, S100), endothelium (GS-IB4), smooth muscle cells (aSMA), and pericytes (NG2, LepR) and surround the vasculature (Zhao et al., 2014; Men et al., 2020), they are also heterogeneous, which reflects the complex functions of MSCs in promoting tissue repair and maintaining tissue homeostasis. ScRNA-seq analysis of mouse incisors showed that Gli1^+^ cells consisted of nine distinct clusters (Chen S et al., 2020). Runx2^+^ cells in the Gli1^+^ MSC subpopulation are not MSCs, and they have been identified as niche cells. These niche cells can regulate the proliferation and differentiation of TACs through IGF signaling (Chen S et al., 2020). In this regard, the role of heterogeneous MSCs in tissue regeneration needs to be reconsidered.

### Axin2^+^ MSCs and Their Role in Odontogenic Regeneration

Axin2, which is expressed in the periosteum and osteogenic Frontier of developing sutures, is a negative regulator of the canonical Wnt pathway (Yu et al., 2005; Di Pietro et al., 2020). Targeted destruction of Axin2 in mice leads to structural malformations of the skull (Yu et al., 2005), suggesting that Axin2 may influence osteoblast proliferation and differentiation.

Axin2^+^ cells were identified in the mouse PDL (Yuan et al., 2018). Severe cementum hypoplasia appeared after the cell ablation of Axin2^+^ cells *in vivo* (Xie et al., 2019), indicating that Axin2^+^ mesenchymal PDL cells may be related to cementum growth.

During the development of alveolar bone, Axin2^+^ cells are activated to form the cementum. After tracing the fate of Axin2^+^ PDL cells in transgenic mice, these cells were found to give rise to the majority of cementoblasts and cementocytes, and the expression of Axin2 in PDL cells was consistent with postnatal cementum growth (Xie et al., 2019; Zhao et al., 2021). Consistently, cell ablation assays showed a significant decrease in the cellular cementum area and acellular cementum thickness of the mandibular molar distal roots after Axin2^+^ cell ablation. In addition, immunostaining revealed that Axin2^+^ cells expressed OSX, a transcription factor in MSCs essential for controlling cementum formation (Cao et al., 2012). Taken together, these findings indicate that Axin2^+^ PDL cells are the major progenitor cell source for both cellular and acellular cementum growth.

Axin2^+^ cells respond to Wnt signaling to promote cementum regeneration. Several studies have suggested that Axin2 is a key target of Wnt/β-catenin signaling, which is a fundamental pathway in many stem cell/regenerative/repair contexts (Lohi et al., 2010; Babb et al., 2017). During the development of the periodontium, both cellular and acellular cementum expanded rapidly, while the level of Wnt signaling decreased gradually (Xie et al., 2019; Men et al., 2020). When roots are fully formed, appropriate Wnt signaling activates Axin2^+^ cells to form cementoblasts to replenish those lost through apoptosis. Thus, maintaining the characteristics of acellular cementum requires low Wnt activity in Axin2^+^ cells.

However, Axin2^+^ cells did not exhibit strong regenerative function after physical injury or infection of periodontal tissue. A likely explanation is that in normally occurring periodontitis the bacteria inhibit the remobilization of Axin2^+^ cells. Moreover, the heterogeneity of Axin2^+^ cells is not well understood. Further research should focus on the mechanism by which Axin2^+^ cells are activated and repressed and the heterogeneity of this specific MSC population to improve clinical treatment outcomes.

### CD24a^+^ MSCs and Their Role in Odontogenic Regeneration

CD24a, also known as heat-stable antigen, is a highly glycosylated molecule with a protein core of only 27 amino acids and is expressed mainly in hamatopoietic and neural cells (Sammar et al., 1997). During development, the expression of CD24a in progenitor cells and metabolically active cells is higher than that in terminally differentiated cells, which reveals that CD24a^+^ cells show cellular pluripotency and have a strong correlation with the self-renewal state (Shakiba et al., 2015). In cancer-related research, it has been proven that CD24a is associated with aggressive tumor behavior, can enhance the tendency of cells to renew, differentiate and metastasize, and can increase the expression levels of enriched Sox2 and Oct4 (Lee et al., 2011).

CD24a + cells were present in both mouse tooth germs and human dental papilla tissues. The CD24a^+^ cell population in human dental papillae is an undifferentiated SCAP population. In response to osteogenic induction conditions *in vitro* culture, the number of CD24a^+^ cells decreased gradually, while the expression of ALP was observed, indicating that CD24a^+^ stem cells may be related to osteogenic differentiation (Sonoyama et al., 2006).

To further study the osteogenic differentiation ability of CD24a^+^ cells, Chen et al. developed a 3D spheroid culture system for tooth-derived stem cells to dissect their lineage commitment and characterize their tissue regenerative potential, which can make up for the deficiency of the traditional two-dimensional (2D) adherent culture system. The results showed that 3D spheroid cultured CD24a^+^ cells maintained their self-renewal state over multiple passages and exhibited enhanced osteogenic/odontogenic differentiation capabilities (Chen H et al., 2020). When transplanted into the renal capsule, these cells could further develop to form regenerative dentin and neurovascular-like structures that mimicked those of native teeth. Therefore, the CD24a^+^ cell population is an excellent alternative cell source for potential translational use in the clinical management of pulpitis and pulp necrosis.

There have been several studies on the mechanisms by which CD24a improves cell proliferation. CD24a has been shown to trigger downstream SRC-family tyrosine kinases, mediating self-renewal and epithelial-to-mesenchymal transition (Sammar et al., 1997; Lee et al., 2011; Lee et al., 2012). Notably, overexpression of CD24a may cause functional inactivation of the tumor-suppressor genes TP53 and ARF, thereby promoting cell proliferation, which provides a link between CD24a and cell growth (Wang et al., 2015). In addition, knockdown of Sp7 was found to strongly abolish the proliferation of CD24a^+^ cells. In this regard, Sp7 may be the key transcription factor driving the self-renewal of CD24a^+^ cells.

However, there have been no *in vivo* trails using the CD24a^+^ cell subpopulation for pulp regeneration to verify its potential for therapy, which was only observed in the renal capsule. In the future, single-cell sequencing will be required to further clarify the composition of the CD24a^+^ cell population and verify its ability to regenerate dental pulp *in vivo*.

### CD146^+^ MSCs and Their Role in Odontogenic Regeneration

CD146, also known as Mel-CAM, MUC18, A32 antigen, and S-Endo-1, is a membrane glycoprotein that functions as a Ca^2+^-independent cell adhesion molecule involved in heterophilic cell–cell interactions (Shih, 1999). CD146 is usually coexpressed with CD73, CD90, CD105, and CD44 on pericytes located outside of capillaries and microvessels in various tissues, such as the muscles, adipose tissue, BM and placenta (Crisan et al., 2008). Several studies suggest that CD146 identifies MSCs with multidirectional differentiation potential (Ma et al., 2021). Compared with CD146^-^ cells, CD146^+^ cells showed the potential to differentiate into adipocytes, chondrocytes, and osteoblasts, while CD146^-^ cells did not (Baksh et al., 2007).

CD146^+^ cells from dental pulp have the ability to generate dentin/pulp-like structures. The coexpression of CD146 and STRO-1 has been shown to be a prerequisite for enhanced stem cell phenotypes, including higher CFU efficiency, expression of embryonic SC markers and enhanced odontogenic differentiation potential (Bakopoulou et al., 2013). In human dental pulp tissue, CD146 and STRO-1 are coexpressed on the cellular membranes of blood vessels (Shi & Gronthos, 2003; Tavangar et al., 2017), indicating that these cells may be a population with enhanced stem cell phenotypes. *In vitro* alizarin red staining and qRT-PCR showed that CD146^+^ cells from dental pulp presented higher mineralization ability than nonseparated cells (Xuan et al., 2018). CD146^+^ cells transplanted into immunocompromised mice also demonstrated their ability to generate dentin/pulp-like structures, compared with CD146^-^ cells and CD146^+/−^ cells (Matsui et al., 2018). Therefore, CD146^+^ cells from dental pulp have the ability to perform mineralization *in vitro*, and to form dentin-like structures *in vivo*. Notably, this characteristic of the CD146^+^ cell subpopulation may be related to the microenvironment required for pulp regeneration. CD146 is a membrane glycoprotein that functions as a Ca^2+^-independent cell adhesion molecule involved in heterophilic cell–cell interactions (Shih, 1999). Several studies have indicated that CD146^+^ cells maintain the hematopoietic microenvironment through the release of paracrine factors, such as vascular endothelial growth factor (VEGF), stem cell factor (SCF), Ang-1 and stromal cell-derived factor (SDF)-1 (Sorrentino et al., 2008), and through cell-to-cell contact mechanisms, such as Notch signaling (Corselli et al., 2013). However, it is not clear whether all or only some CD146^+^ cells are involved in the maintenance of microenvironmental homeostasis. Further subdivision of CD146^+^ cells is needed to clarify their role in pulp regeneration.

In addition, CD146^+^ cells in the PDL also have the capacity to generate a cementum/PDL-like structure and contribute to periodontal tissue repair. Seo et al. isolated STRO-1^+^/CD146^+^ cells from PDLSCs for the first time, showing their capacity to develop into cementoblast-like cells, adipocytes *in vitro*, and cementum/PDL-like tissue *in vivo* (Seo et al., 2004). Impressively, PDLSCs also showed the capacity to form collagen fibers, similar to Sharpey’s fibers, connected to the cementum-like tissue, suggesting their potential to regenerate PDL attachment. However, the limited number of the PDLSCs in extracted teeth limits their application in periodontal tissue regeneration. To obtain sufficient cell numbers, expansion of PDLSCs by cell culture with growth factors such as FGF-2 is essential (Hidaka et al., 2012).

Taken together, these findings reflect the fact that CD146^+^ cells exhibit a remarkable capacity for regeneration. However, *in vitro* culture for amplification may increase the heterogeneity of the CD146^+^ cell population, which may affect clinical application. Further research needs to be done to investigate the respective roles of different subpopulations of CD146^+^ cells in tissue regeneration, and to explore how to increase the number of cells on a large scale without affecting their functions.

### STRO-1^+^ MSCs and Their Role in Odontogenic Regeneration

STRO-1 is considered the best-known marker used to identify MSCs (Kolf et al., 2007). STRO-1 is a 75 kDa endothelial antigen that is localized to the endothelium of some arterioles and capillaries in some tested tissues, such as adipose tissue, muscle, liver, lungs and kidneys (Lin et al., 2011; Ning et al., 2011). STRO-1-negative cell populations have been reported to be unable to form colonies (Simmons and Torok-Storb, 1991). Only cells expressing the STRO-1 antigen are capable of becoming hematopoiesis-supportive stromal cells with a vascular smooth muscle-like phenotype, adipocytes, osteoblasts and chondrocytes (Dennis et al., 2002), which is consistent with the functional role of MSCs.

STRO-1^+^ cells isolated from human adult dental pulp were located in the perivascular region (Shi and Gronthos, 2003). It was reported that sorted STRO-1^+^ dental pulp cells demonstrated the capacity to form multiple layers and mineralized nodules when cultured in conditioned medium, whereas the STRO-1^-^ population showed a lack of the capability to form mineralized nodules *in vitro* (Yang et al., 2007). Thus, STRO-1^+^ cells might represent a better source of cells for therapeutic purposes than unsorted heterogeneous cells.

STRO-1^+^ cells exhibit greater clonogenicity, proliferative capacity and multilineage differentiation potential than STRO-1^-^ or the nonsorted cell population (Simmons and Torok-Storb, 1991; Gronthos et al., 2001; Psaltis et al., 2010). STRO-1^+^ cells were observed to achieve approximately 20% more population doublings than nonsorted cell populations *in vitro* culture, ultimately resulting in higher cumulative population expansion *in vitro* (Gronthos et al., 2003; Psaltis et al., 2010). In addition, STRO-1^+^ cells consistently exhibited higher mRNA transcript levels, which have been associated with early mesenchymal development and/or proliferative capacity (Psaltis et al., 2010). This may explain the high cloning efficiency of STRO-1^+^ cells. Notably, it was suggested that the telomerase activity of STRO-1^+^ cells was greatly enhanced (Simonsen et al., 2002), whereas the telomerase activity of normal somatic cells was lost during proliferation and differentiation (Fuchs and Segre, 2000). Telomerase activity is a key factor oin the proliferative lifespan and osteogenic potential of human BM mesenchymal cells in culture. Therefore, the enhanced telomerase activity of STRO-1^+^ cells also greatly enhanced their proliferative and osteogenic potential.

STRO-1^+^ cell sorting improves the purity of MSCs, which may affect their odontogenic differentiation capacity. However, it was recently found that although the proportion of STRO-1^+^ cells expanded during osteogenic differentiation (Perczel-Kovach et al., 2021), they did not show a better performance than STRO-1^-^ or unsorted heterogeneous cells in osteogenesis (Gurel Pekozer et al., 2018). This is probably because STRO-1 simply distinguishes a subpopulation of MSCs that are unique in terms of adherence, proliferation and multilineage differentiation potential, but these cells are still heterogeneous. Therefore, further studies are needed to determine the composition of STRO-1^+^ cells and to determine which subpopulation has stronger odontogenic differentiation ability.

### CD105^+^ MSCs and Their Role in Odontogenic Regeneration

CD105 (Endoglin, SH2), a 180 kDa homodimeric glycoprotein, is a component of the TGF-β receptor complex and is mainly found on circulating vascular endothelial cells in regenerating, inflamed tissues or tumors with active angiogenesis (Cheifetz et al., 1992). Nakashima et al. isolated a CD105^+^ cell population from human adult dental pulp tissue for the first time by flow cytometry and demonstrated colony formation activity of these cells (Nakashima et al., 2009).

Compared with total pulp cells, CD105^+^ pulp cells showed high proliferation and migration activities and expressed higher levels of glial cell line-derived neurotrophic factor (GDNF) and VEGF-A *in vitro*, suggesting that they appear to have good cell properties for pulp regeneration.

The CD105^+^ cell subpopulation may be involved in nerve and vascular development. CD105 has been associated with active angiogenesis (Cheifetz et al., 1992). CD105^+^ cells showed multilineage differentiation potential, including adipogenesis, dentinogenesis, angiogenesis and neurogenesis potential, *in vitro* (Iohara et al., 2011). In an experimental model of tooth autogenous transplantation of CD105^+^ cells together with type I and type III collagen as a scaffold, pulp tissue achieved complete regeneration of capillaries and neuronal processes, whereas unsorted DPSCs were preferable for differentiation into odontoblasts to mineralize and form dentin in the pulp chamber and root canal (Nakashima et al., 2009). Thus, compared with total pulp cells, the pulp CD105^+^ cell subpopulation may be a candidate cell resource for the induction of pulp regeneration by cell therapy.

Moreover, CD105^+^ cells are capable of trophic effects on endothelial cells. It was reported that the transplanted CD105^+^ cells were likely to be located near the newly formed vasculature and expressed proangiogenic factors, implying their nutritional effect on the formation of new blood vessels (Iohara et al., 2011).

However, the CD105^+^ cell subpopulation is inherently heterogeneous, and some fractions, such as the CD105^+^/STRO-1^-^ population, may have a negative effect on the differentiation potential of the whole population (Gothard et al., 2014). Further subdivision of the CD105^+^ cell population is needed in the future to achieve a stable therapeutic effect in the context of pulp regeneration.

## Conclusion and Perspective

The body of work outlined in this review highlights the biological characteristics of several odontogenic MSC subpopulations. Recent studies suggest that odontogenic MSCs have enormous potential in the regeneration of pulp, periodontium and alveolar bone. However, the phenotypic and genetic differences among cell subpopulations affect the function of MSCs. There are also differences in proliferation and differentiation potential among stem cells isolated from the same site, which makes their role in tissue regeneration and disease management unclear, posing a challenge for the development of regenerative medicine. In addition, different cell subpopulations also provide different treatment approaches for diseases. It is essential to further clarify the biological differences between odontogenic MSC subpopulations and evaluate their therapeutic benefits in specific tissue regeneration contexts in the field of regenerative medicine. It may be important to improve the outcomes of regenerative therapy via cell sorting, which aims to divide cells into subpopulations according to their functional characteristics, and subsequent application of one or more subpopulation for different needs. To achieve this, larger patient-donor cohorts need to be included, and lineages of MSC subsets with different proliferation and differentiation characteristics need to be studied more extensively using extensive genomic and proteomic sequencing to explore more reliable gene, protein and metabolic markers.

## Abbreviations

2D, two-dimensional; ALP, alkaline phosphatase; BMSCs, bone marrow stromal cells; DFSCs, dental follicle stem cells; DP, dermal papilla; DPSCs, dental pulp stem cells; GDNF, glial cell-line derived neurotrophic factor; GMSCs, gingival mesenchymal stem cells; Hh, hedgehog signaling; hDPSCs, the human deciduous pulp stem cells; hPCy-MSCs, human periapical cyst-mesenchymal stem cells; MSC, mesenchymal stem cell; PA-MSC, plastic adherence isolated MSC; PDL, periodontal ligament; PDLSCs, periodontal ligament stem cells; SCAPs, stem cells of the apical papilla; SCF, stem cell factor; scRNA-seq, single-cell RNA sequencing; SDF-1, stromal cell-derived factor; SHED, Stem cells from exfoliated deciduous teeth; Th17, T helper 17; Tregs, regulatory T cells; VEGF, vascular endothelial growth factor.
